# *In vivo* T2* weighted MRI visualizes cardiac lesions in murine models of acute and chronic viral myocarditis

**DOI:** 10.1371/journal.pone.0172084

**Published:** 2017-03-06

**Authors:** Xavier Helluy, Martina Sauter, Yu-Xiang Ye, Gunthard Lykowsky, Jakob Kreutner, Ali Yilmaz, Roland Jahns, Valerie Boivin, Reinhard Kandolf, Peter M. Jakob, Karl-Heinz Hiller, Karin Klingel

**Affiliations:** 1 Department of Experimental Physics V, Institute of Physics, University of Wuerzburg, Wuerzburg, Germany; 2 Department of Molecular Pathology, Institute for Pathology and Neuropathology, University Hospital Tuebingen, Tuebingen, Germany; 3 Center for Systems Biology, Massachusetts General Hospital and Harvard Medical School, Boston, United States of America; 4 Research Center Magnetic-Resonance-Bavaria (MRB), Wuerzburg, Germany; 5 Department of Cardiology and Angiology, University Hospital Münster, Münster, Germany; 6 Comprehensive Heart Failure Centre (CHFC) and Interdisziplinary Bank of Biomaterials and Data (ibdw), University Hospital of Würzburg, Würzburg, Germany; 7 Institute of Pharmacology and Toxicology, University of Würzburg, Würzburg, Germany; 8 Fraunhofer Institute for Integrated Circuits, Magnetic Resonance and X-Ray Imaging Department, Würzburg, Germany; Scuola Superiore Sant'Anna, ITALY

## Abstract

**Objective:**

Acute and chronic forms of myocarditis are mainly induced by virus infections. As a consequence of myocardial damage and inflammation dilated cardiomyopathy and chronic heart failure may develop. The gold standard for the diagnosis of myocarditis is endomyocardial biopsies which are required to determine the etiopathogenesis of cardiac inflammatory processes. However, new non-invasive MRI techniques hold great potential in visualizing cardiac non-ischemic inflammatory lesions at high spatial resolution, which could improve the investigation of the pathophysiology of viral myocarditis.

**Results:**

Here we present the discovery of a novel endogenous T2* MRI contrast of myocardial lesions in murine models of acute and chronic CVB3 myocarditis. The evaluation of infected hearts *ex vivo* and *in vivo* by 3D T2w and T2*w MRI allowed direct localization of virus-induced myocardial lesions without any MRI tracer or contrast agent. T2*w weighted MRI is able to detect both small cardiac lesions of acute myocarditis and larger necrotic areas at later stages of chronic myocarditis, which was confirmed by spatial correlation of MRI hypointensity in myocardium with myocardial lesions histologically. Additional *in vivo* and *ex vivo* MRI analysis proved that the contrast mechanism was due to a strong paramagnetic tissue alteration in the vicinity of myocardial lesions, effectively pointing towards iron deposits as the primary contributor of contrast. The evaluation of the biological origin of the MR contrast by specific histological staining and transmission electron microscopy revealed that impaired iron metabolism primarily in mitochondria caused iron deposits within necrotic myocytes, which induces strong magnetic susceptibility in myocardial lesions and results in strong T2* contrast.

**Conclusion:**

This T2*w MRI technique provides a fast and sensitive diagnostic tool to determine the patterns and the severity of acute and chronic enteroviral myocarditis and the precise localization of tissue damage free of MR contrast agents.

## Introduction

Viral myocarditis is a major cause of heart failure and sudden death in young adults [1–3]. The gold standard for the diagnosis of viral myocarditis relies on endomyocardial biopsies, the only aetiopathogenic approach with, however, limited sampling coverage of the heart [4,5]. Cardiac MRI detects necrotic or fibrotic lesions in acute and chronic myocarditis using late gadolinium enhancement (LGE) T1 weighted contrast technique [6]. This non-invasive technique still requires contrast agent injection and dynamic MR image acquisition.

The pathogenesis and the underlying molecular mechanisms of acute and chronic myocarditis and resulting dilated cardiomyopathy have been mostly studied in animal studies [7]. In particular, coxsackievirus B3 (CVB3)-induced chronic myocarditis in susceptible mice such as ABY/J and SWR/J strains are most important mouse models reflecting very well different stages of viral myocarditis in patients [8]. However, only recently the first applications of cardiac MRI have been presented in this model in order to improve the non-invasive diagnosis of viral myocarditis. Brunner et al. [9] used cine MRI to measure alterations of cardiac function parameters. Jacoby et al. [10] and Ye et al. [11] demonstrated imaging of macrophage infiltration by using ^19^F fluorine MRI tracers in experimental myocarditis models.

In this work, we pursue to improve the non-invasive diagnosis of myocarditis by MRI techniques in the murine model of acute and chronic CVB3 myocarditis and report on the discovery of an unexpected endogenous MRI contrast using standard T2* weighted MRI. The evaluation of this contrast allowed a direct localization of virus-induced cardiac lesions without any MRI tracer or contrast agents, and thus to correlate the outcome and severity of myocarditis. Additional MR image analysis and histopathological investigations were performed in order to identify the mechanisms of the reported MR contrast and its biological origins. Finally, we report on the difficulty of using an iron oxide based contrast agent, ferumoxytol, to visualize inflammatory lesions in this animal model of CVB3-induced myocarditis.

## Material and methods

### Virus and animals

All procedures and animal studies were approved by the Regierungspräsidium Tübingen (permission no. PA1/08). Four-week-old mice from two mouse strains (SWR/J (H-2q) and ABY/SnJ (H-2b)) were infected with CVB3 Nancy strain as described previously [12,13]. Non-infected animals of both strains served as controls. For *ex vivo* MRI and histology, hearts were taken and perfused with PBS to remove blood from myocardium in order to avoid residual blood causing signal voids on *ex vivo* T2 and T2* weighted images. The hearts were fixed in 4% buffered formaldehyde for one day and further preserved in PBS at 4°C. Experimental groups of CVB3-infected ABY/SnJ and SWR/J mice (n = 3–6 per group) were used for *ex vivo* or *in vivo* MRI measurements at different time points post infection (p.i.) (6 days, 9 days for acute myocarditis, 14 days for sub-acute myocarditis and eight weeks and more for chronic myocarditis). Table 1 summarizes the different groups of animals investigated by MRI and histology with exact number of animals per group. The rationale driving the number of animals per group was the following: in this animal model of myocarditis the exact microscopic patterns of lesions are well known. There are only three groups of morphological findings: (no: not-infected control mice(H1), mild (H2) and severe myocarditis (H3)) we see at any time point post infection. With 5 infected animals per group we always observe animals with mild and severe myocarditis in each group at all time points post infection. Thus, each of these 3 groups forms an entity regarding the comparison of MRI findings with histology.

**Table 1:** Animal assignments and comparisons of MR and histology for all sets of animals investigated.

Animal Set	Time After Infection	Mouse strain	*In vivo Ex vivo*		*MR1*	*MR2*	*MR3*
A	Chronic	SWR/J	*in vivo*	***H1***	0	0	0
	2–10 months			***H2***	0	0	0
Pre study				***H3***	0	0	**3**
Animals selected			*ex vivo*	***H1***	0	0	0
from larger group for				***H2***	0	0	0
strong T2* abnormalities				***H3***	0	0	**2**
B	Chronic	SWR/J	*in vivo*	***H1***	**2c**	0	0
	15 weeks			***H2***	**2**	**1**	0
				***H3***	0	0	**1**
C	Chronic	ABY	*in vivo*	***H1***	**3c**	0	0
	8 weeks			***H2***	**1**	**1**	0
				***H3***	0	0	**3**
			*ex vivo*	***H1***	**2c**	0	0
			subset from	***H2***	**1**	**1**	0
			*in vivo*	***H3***	0	0	**3**
D	Acute	ABY	*in vivo*	***H1***	**0**	0	0
	14 days			***H2***	2	**3**	0
				***H3***	0	0	**3**
E	Acute	ABY	*ex vivo*	***H1***	0	0	0
	14 days			***H2***	0	**3**	0
				***H3***	0	0	**2**
F	9 days	SWR/J	*ex vivo*	***H1***	**2c**	0	0
				***H2***	**1**	**1**	0
				***H3***	0	0	**1**
G *	9 days	ABY	*ex vivo*	***H1***	0	0	0
				***H2***	0	**1**	0
				***H3***	0	0	**4**
H	6 days	SWJ	*ex vivo*	***H1***	**0**	0	0
				***H2***	**2**	**2**	0
				***H3***	0	0	0
I	6 days	ABY	*ex vivo*	***H1***	**0**	0	0
F+G+H+I inoculated				***H2***	**2**	**3**	0
at same time				***H3***	0	0	**1**

* one animal was excluded. It was giving strong abnormal contrast ex vivo but not in histology. The analysis revealed a technical error in histological workup. The abbreviations MR1, MR2 and MR3 are used to designate the 3 defined MR groups: MR1 no myocarditis, MR2 mild myocarditis and MR3 severe myocarditis. The abbreviation H1, H2 and H3 are used to define the histological groups no myocarditis (H1, observed only in control animals), mild myocarditis (H2) and severe myocarditis (H3). We observed non-zero values only along the diagonals in the case of severe myocarditis, meaning that no mismatch between histology and MR diagnostic in animals with severe myocarditis was found. Note that some animals with a mild myocarditis are not detected by MRI (animals on off-diagonal) due to the higher sensitivity of histology which is capable of detecting myocyte damage at the single cell level compared to MRI. Those animals represent always cases with little numbers of very small lesions. Numbers followed by letter c indicate non-infected control mice.

In order to investigate whether superparamagetic iron-oxide nanoparticles can be used for the visualization of immune cells (macrophages) in the heart of CVB3-infected mice by MRI, animals were subjected to tail vein injections of a 10 or 40 mg Fe/kg ferumoxytol (Feraheme, AMAG Pharmaceuticals, Lexington, MA) 12 days p.i. Two days after the ferumoxytol injection, the animals were remeasured by MRI and hearts were later investigated by histology.

### Magnetic resonance imaging

#### *Ex vivo* MRI at high magnetic field

*Ex vivo* 3D T2w (7 hours acquisition, 50x100x100 μm voxel size) and T2*w (with 100 μm isotropic resolution) were acquired on a vertical Bruker AMX 500 microscopy system at 11.7 T with a maximum gradient strength of 660 mT/m using a 500.15 MHz 1H transceive quadrature birdcage resonator (RAPID Biomedical, Germany). Commercially available 3D spin echo and 3D spoiled multi-gradient echo sequences (Paravision 4.0 software) were used. Additional parameters for T2* weighted images: Matrix size 128x100x100, repetition time TR = 400 ms, echo times TE [ms] = 3, 6.5, 10, 13.5, 17, 20.5, 24, and 27.5. Frequency encoding = 13.2 kHz, bandwidth = 591.8 Hz/voxel, radio-frequency selective pulse bandwidth = 2700 Hz, flip angle = 50°, total acquisition time was 6h40min using 6 averages. Spin echo sequence parameters: TR = 2.5 s, TE = 14 ms, acquisition time = 7 hours, frequency encoding bandwidth = 195.3 Hz/voxel, radio-frequency selective pulse bandwidth = 2.7 kHz. Moreover, additional hearts were imaged with an ultra-high resolution of 50 *μm* isotropic using the AMX-500 system and a spoiled gradient echo sequence (isotropic resolution) or a vertical Bruker Avance 17.6 T microscopy system with a maximum gradient strength of 1 T/m. A commercially available 15 mm birdcage resonator was used. Two infected hearts were finally measured with an ultra-high resolution of 20 *μm* at 17.6 T using a scroll coil [14] built in-house (S2 Movie). Additionally, *ex vivo* imaging was performed directly from hearts immersed in 4% buffered formaldehyde. A comparison of image contrast on hearts affected by severe myocarditis imaged in 4% formaldehyde solution or washed in PBS after fixation did not reveal any difference concerning the reported observed T2* hypointense signals.

#### *In vivo* MRI at high magnetic field

*In vivo* MRI was performed on non-infected control mice and non-contagious CVB3-infected mice from day 14 p.i. up to 10 months after CVB3 infection. Breath gated and ECG triggered multi-slice multiple spoiled gradient-echo short axis heart images were acquired on a preclinical Bruker Biospec 7 T scanner, using an 870 mT/m gradient system and a commercial transmit/receive quadrature birdcage coil with inner diameter 3.5 cm. Seven to eleven slices of images were acquired in single slice and/or interleaved multi-slice modus. Single slice images were acquired in addition to multi-slice images to increase image quality of slices of interest thanks to reduced sensitivity to blood flow artifact and increased contrast between myocardium and blood. Echo times were: 2.6 ms, 6 ms, 9.4 ms, 12.8 ms, 16.2 ms. FA = 60–80° when multislice acquisition with 7–11 slices, FA = 20° for single slice acquisition. Data were averaged 4–8 times but such as acquisition time would not exceed 20 min. Typical matrix size = 256x170 points, FOV = 3x2 cm, slice thickness = 0.5 mm. RF Pulse interval = heart period.

#### *Ex vivo* MRI investigation at clinical static magnetic field strength

Also, formalin-fixed hearts from ABY/SnJ mice with severe myocarditis obtained 9 days p.i. (as diagnosed using T2*w images at 7 T) were imaged *ex vivo* using the preclinical 17.6T system (TE = 16 ms) and a clinical 1.5T scanner (SIEMENS Avento, TE = 18 ms). Commercial 3D spoiled gradient echo sequences were used with imaging parameters as similar as possible between the two scanners. In particular, voxel size was 146 μm and bandwidth was 120 Hz/voxel.

### Histology

Six 1 mm thick slices along the heart long axis obtained by using a tissue slicer were embedded in paraffin. Axial tissue sections (5 μm) were cut and stained with hematoxylin/eosin (HE) and Masson’s trichrome stain to assess myocardial injury, inflammation and fibrosis or with Prussian blue to assess iron deposits (Fe^3+^) as described previously [13]. In addition, calcification was examined by von Kossa staining. For the qualitative histological assessment of myocardial damage and definition of grades of myocarditis infection only sections distal to atria were considered. Infected animals with sections presenting very few or sparse small lesions were assigned a grade of mild myocarditis (H2). Animals with numerous lesions had severe myocarditis (H3). Animals without any visible lesions (observed in control animals only) were assigned the grade H1 (no lesion, i.e. non-infected control animals).

### Electron microscopy

Heart tissue samples were fixed in 3% glutaraldehyde in 0.2 M phosphate buffer (pH 7.4) for 1 hour at room temperature. After three washes for 1 hour at room temperature in 0.2 M phosphate buffer, the tissue slices were fixed in 1% osmium tetroxide in the same buffer, dehydrated in a graded series of ethanol, blocked-stained with 1% uranylacecate and 1% phosphotungstic acid in 70% ethanol, further dehydrated and embedded in Araldite (Merck, Darmstadt, Germany). Ultrathin sections (70 nm) were cut with a diamond knife on an ultramicrotome and picked up on 300 mesh nickel grids. Sections were examined with a Zeiss EM 902 transmission electron microscope at a magnification of 5,000–30,000.

### Identification of T2* hypointense signals in *in vivo* or *ex vivo* cardiac MRI

**Fig 1:**
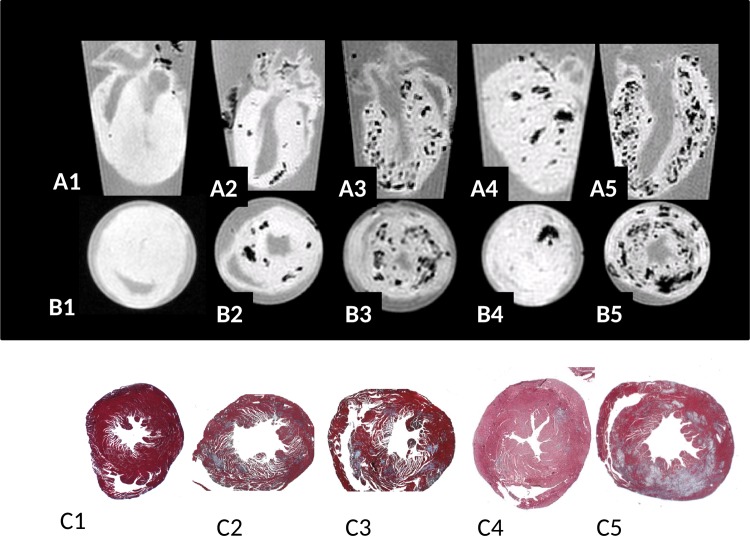
Long axis (row A) and short axis (row B) T2* weighted (TE = 3 ms, 100 μm isotropic resolution) images of *ex vivo* hearts from CVB3-infected ABY/SnJ mice during acute myocarditis. Corresponding Masson's trichrome stained heart tissue sections (short axis) from (1) non-infected control mouse, (2) mild acute myocarditis (9 d p.i.), (3) severe acute myocarditis (9 d p.i.), (4) mild subacute myocarditis (14 d p.i.) and (5) subacute severe myocarditis (14 d p.i.). The extent and location of of hypointense regions seen in T2* weighted MRI images (row B) agree well with patterns of cardiac of lesions as visualized by Masson's trichrome staining (row C, x5 magnification).

Images were analyzed by experienced researchers in the field of small rodents cardiac MRI and a radiologist. Care was taken to separate abnormal T2* hypointense signals from more common sources of signal losses in CMR images of mice induced for example by lung susceptibility inhomogeneity, blood flow, motion artifacts etc. To mitigate such artefacts, additional images were acquired in case of doubt on the presence or absence of abnormal T2* contrast. In case of e*x vivo* images, we qualified a T2* hypointense signal as abnormal, when it could not be associated with high confidence to air bubbles or remaining blood clots in heat chambers or large vessels. For clarity, we always write hypointense signals or dark spots to specifically refer to abnormal T2* signals only, reverting to the exact denomination only if requested by context. For all infected animals, investigators were blinded both at MR acquisition and at MR analysis since the degree of disease was unknown at scan time and during MR evaluation. Depending on the amount and size of the lesions observed by MRI, animals were assigned to one of the following 3 MR diagnostic groups: no myocarditis (no sign of abnormal T2* contrast, called MR1 grade), mild myocarditis (at least one well defined lesion in one slice, see examples in Fig 1 hearts 2 and 4), called MR2 grade or severe myocarditis, called MR3 grade (numerous lesions or at least one very large hypointense region, see Fig 1 hearts 3 and 5). After histological evaluation, the MR diagnostic was correlated with the histological reference.

#### Correlation of histological sections with 2D *in vivo* MR images

In this study, we performed a matching of *in vivo* MR images with the histological tissue sections, considering that the matching is only approximate due to the non-local nature of the T2* contrast, the non-continuous sectioning of the tissue slices as well as due to the differences in histological and MR slice thickness, in-plane resolution and orientation. Furthermore, tissue fixation and shrinking due to paraffin embedding and sectioning further influences the depiction between hearts imaged *in vivo* with MR and heart tissue as seen in histological sections.

#### Correlation of histological sections with 3D *ex vivo* MR images

For all investigated isolated hearts *ex vivo*, 3D MRI datasets as well as selected digitized histology slices (Masson’s trichrome and Prussian blue stained sections) were imported into the commercial 3D software Amira (FEI. Visualization Sciences Group, Burlington, MA, USA). Histological sections were displayed in a separate pane from the MRI data. After optimization of the MR image contrast between buffer and myocardium, the position and orientation of oblique slices within the 3D MR dataset were manually adjusted in order to give optimal visual matching between pathological as well as non-pathological structures observed with MRI and histology.

## Results

### *Ex vivo* T2* contrast study of acute myocarditis at high static magnetic field

Hearts of SWR/J and ABY/SnJ mice, both susceptible for chronic myocarditis were investigated at different time points after CVB3 infection by *in vivo* and *ex vivo* MRI, demonstrating comparable results. Our initial *ex vivo* MRI study at 11.7 T revealed an unexpected patchy T2* (TE = 3 ms) hypointense contrast of CVB3-infected myocardium in both mouse strains at any investigated time point p.i., which was not observed in uninfected hearts (Fig 1 heart 1). Typical areas with patchy hypointense T2* contrast during acute myocarditis (day 9 and 14 p.i.) are exemplarily shown in Fig 1, heart 2–5, long axis row A, short axis row B of ABY/SnJ mice. The frequency and localization of the observed T2* hypointense regions correlated well with virus-induced inflammatory lesions as detected by Masson's trichrome stainings obtained from the same regions of the hearts (Fig 1, row C). The different patterns of virus-induced myocardial lesions are well reflected by the T2*w MRI images, thus allowing in particular to visualize the outcome and severity of acute myocarditis. A typical example of mild myocarditis is shown in Fig 1, heart 4, and of severe myocarditis in Fig 1, heart 5, both day 14 p.i. The grade of myocarditis, as revealed by histology, is systematically compared to the MR diagnostic in Table 1.

### Identifying the biological cause of hypointense regions in acute myocarditis

It is well known that pathological T2*w hypointense regions in MRI are suggestive for being paramagnetic. One well-defined atomic/molecular carrier of paramagnetic inhomogeneities is given by endogenous (super) paramagnetic iron deposits [15]. Recently, we have observed significant iron accumulations in infected myocytes from CVB3-induced cardiac lesions of susceptible SWR/J mice [13] during acute as well as chronic myocarditis.

**Fig 2:**
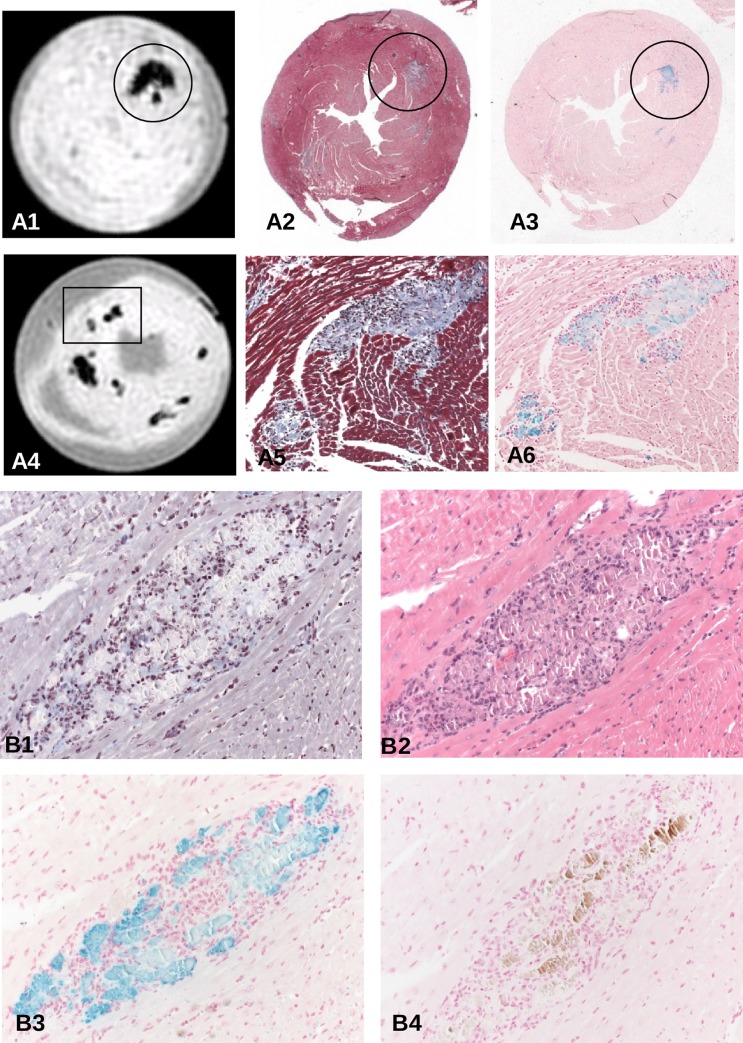
(A) Spatial correlation of T2*w images of hearts 4 (A4-6, 9 days p.i.) and 2 (A1-3, 14 days p.i.) of Fig 1 with Masson's trichrome and Prussian blue-stained lesions. Iron deposits as stained by Prussian blue can be attributed to inflammatory lesions, mainly to affected cardiomyocytes at any time of acute and subacute myocarditis (A2, A3, x6; A5, A6 reflect area covering insert of A4, x200). (B) Dissection of an individual cardiac lesion by different histopathological stainings in consecutive tissue sections from a ABY/SnJ mouse 14 days p.i. (B1, Masson's trichrome, B2, HE, B3 Prussian blue, B4 von Kossa stain). Note, that the virus-induced damaged myocytes demonstrated in B1 and B2 spatially correspond well to iron deposits in myocytes (B3) whereas calcification (brown) occurs only in a part of the affected myocytes (B4) (x200).

In order to evaluate whether the hypointense regions in MRI are due to iron incorporation we stained consecutive tissue sections by HE, Masson's trichrome and Prussian blue for all investigated hearts. We noted that iron is present in acute and chronic myocarditis as early as day 6 p.i. in inflammatory lesions of SWR/J mice and also of ABY/SnJ mice. Fig 2A illustrates the close spatial correlation of hypointense regions in MRI T2*w images (A1 and A4) with inflammatory lesions (Masson's trichrome, A2 and A5) and iron (Prussian blue staining, A3 and A6) in the hearts of two ABY/SnJ mice demonstrated in Fig 1 (heart 2, day 9 p.i. (A4-6) and heart 4, day 14 p.i. (A1-3) respectively). The variability in the intensity of Prussian blue staining demonstrates different concentrations of iron in infected and necrotic myocytes, which are dependent on the stage and progress of virus-induced damage. However, independent to the amount of iron we find MRI hypointensity at all stages of myocarditis (see additional observations in section on additional MRI results). In order to exclude that fibrosis or calcification is responsible for hypointensity effects in MRI we stained consecutive tissue sections of mouse hearts with Masson's trichrome, HE, von Kossa and Prussian blue. As exemplarily shown in Fig 2B the area fraction of calcium precipitates (brown, B4) or collagen (blue, B1) present in cardiac lesions is very low compared to the extent of iron deposits (blue, B3) within inflammatory lesions (B2) in the heart obtained 14 days p.i. Thus, the close spatial relationship of iron positive areas with hypointensity areas in MRI at any stage of myocarditis provides firm evidence that iron is the decisive element in the detection of cardiac lesions in our mouse model by MRI. Blood (mircobleeds) as cause of iron deposition was excluded by our histological staining.

### Evaluation of CVB3 infection by transmission electron microscopy

**Fig 3:**
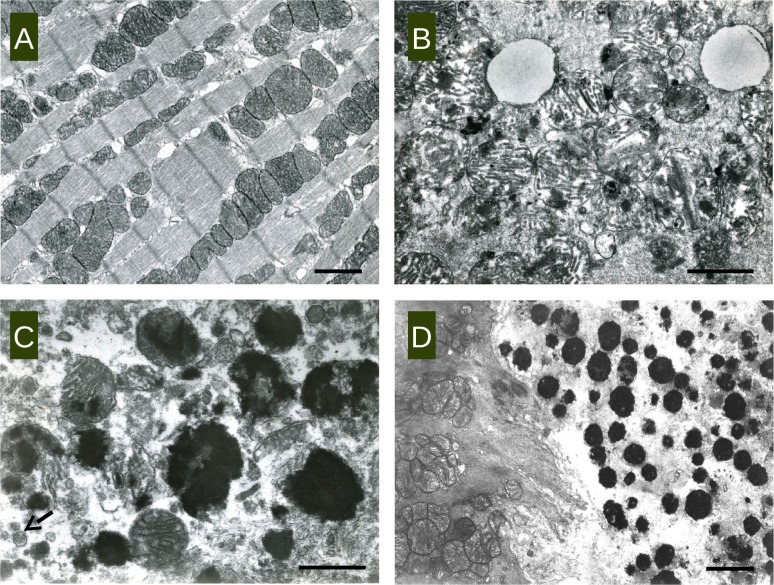
Transmission electron microscopic image of representative heart tissue samples of ABY/SnJ mice (A) 0 days p.i. (uninfected control), (B) 6 days p.i., (C) 9 days p.i. and (D) 28 days p.i. Deposits of electro-dense material in mitochondria in association with structural disturbances of mitochondria and the cytoplasm of myocytes are observed at any stage of infection. (C arrow: virions; A, bar: 2 μm, B bar:1 μm, C bar:1,4 μm, D bar:2,5 μm).

In order to evaluate iron deposition observed in Prussian blue staining in more detail we examined representative tissue samples of CVB3-infected murine hearts from ABY/SnJ mice by transmission electron microscopy in the course of infection. In contrast to non-infected control hearts (Fig 3A) we found electron dense deposits in myocytes at any stage of infection (Fig 3B–3D). As shown in Fig 3B, at 6 d p.i. small areas with electron dense material are primarily observed in mitochondria. Starting from day 9 p.i. there is a considerable increase in the amount of electron dense material in mitochondria (Fig 3C) primarily in the infected (arrow illustrates virions) and damaged myocytes. In the final stages of infection (Fig 3D, 28 days p.i.), the virus-induced necrotic myocyte (right side of image) contains plentiful electron dense deposits, consistent with iron. On the left side, an uninfected myocyte is demonstrated without any kind of deposits. Importantly, deposits were found to be consistently associated with structural alterations of mitochondria, namely of disruption of cristae and, in parallel with severe disturbances in the myocyte architecture. Infected myocytes (arrow points to virions) with many electron dense deposits revealed a severe loss of myofibrils in absence of normal mitochondria (Fig 3D, right side), reflecting necrosis typically observed in CVB3-infected mice as shown in Fig 1.

### *Ex vivo* T2* contrast study of chronic myocarditis at high static magnetic field

**Fig 4:**
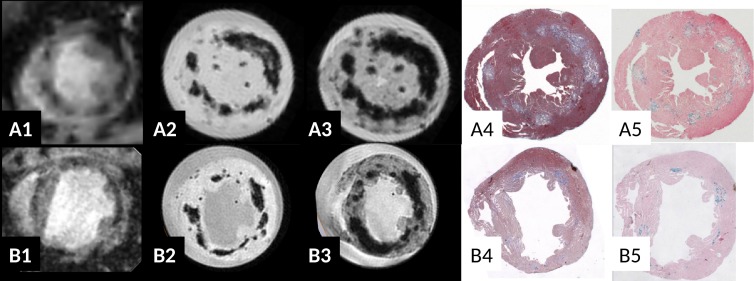
Correlation of T2* w contrast *in vivo* (A1, B1: TE = 2.6ms) and *ex vivo* (A2, B2 TE = 2.6/6ms) in chronic myocarditis in ABY/SnJ mice (8 weeks p.i., row A) and SWR/J mice (8 months p.i., row B). The location of the T2* hypointense regions correlates very well with virus induced lesions as observed in Masson's trichrome staining (A4, B4) and Prussian blue staining (iron deposits, A5, B5). An increase in T2* blooming effect in *ex vivo* images is found at longer echo times. Note severe dilation of the left ventricle 8 months p.i. indicating DCM and heart failure in the SWR/J mouse 8 months p.i. (B).

In addition to hearts obtained during acute infection, mouse hearts of 8 weeks post infection up to several months after CVB3 infection were investigated by *ex vivo* T2*w MRI. As demonstrated in both ABY/J (Fig 4A, 8 weeks p.i.) and SWR/J mice (Fig 4B, 8 months p.i.), strong hypointense regions in myocardium were observed in MRI at late stages of myocarditis. The frequency and location of the T2* dark spots correlated very well with the grade of the CVB3-induced myocardial lesions (Fig 4A4 and 4B4). Prussian blue staining of myocardial lesions was generally strong (Fig 4A5 and 4B5), but appeared, as already seen during acute infection, sometimes inhomogeneous (Fig 4A5). As shown in cases with short echo times (Fig 4A2), the shape of the hypointense regions seem to follow the periphery of the cardiac lesions. This suggests that the T2* contrast *ex vivo* correlates well with the distribution of iron as identified within Prussian blue staining. Altogether, the findings in MRI revealing hypointense regions are rather similar in hearts with acute or chronic myocarditis.

### T2* blooming demonstrates existence of strong local magnetic susceptibility inhomogeneities

**Fig 5:**
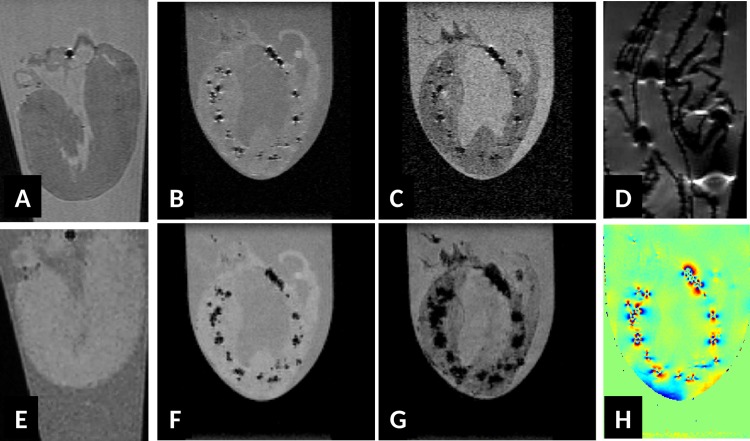
*Ex vivo* T2* weighted (E-F, TE = 3ms; G, TE = 10ms) and T2 weighted (A-B, TE = 10ms; C, TE = 30ms, BW = 50kHz) long axis magnitude images of isolated hearts from SWR/J mice (control animals (A and E) and with severe cardiomyopathy (same animal as in Fig 4B), eight months after infection (B, C, F, G). Note the strong blooming effect of hypointense regions only observable in T2*w images with increasing echo time (compare G to F) and not in T2w images (compare C to B) (B, C, F, G). Image H is the phase image of the infected myocardium associated to the T2*w magnitude image F. Strong phase distortions appear inside or in close proximity to cardiac lesions. D: magnitude T2w image of a 4% formalin phantom containing air bubbles trapped inside folded sheet of paper. Same frequency encoding direction used for phantom imaging and T2 weighted images of isolated myocardium in A-C. Note the similarity of the “arrowhead”-like structures with hyperintense signals and hypointense regions induced by air bubbles or cardiac lesions in T2 weighted images (compare D with B, C). Those similar patterns indicate that damaged tissue is more paramagnetic than healthy myocardium. Colored inset in Fig A represents the phase image of the observable air bubble or blood clot in control heart A.

On increasing echo time, the size of the reported hypointense regions increased in all recorded *ex vivo* T2*w gradient echo images and for all hearts where cardiac lesions were visible, as illustrated in Fig 4A2 (short TE), A3 (long TE), B2 (short TE), B3 (long TE) and in Fig 5F (short TE) and G (long TE). From a physical point of view, this so called “T2* blooming effect” indicates the presence of local magnetic susceptibility inhomogeneities in the vicinity of the cardiac lesions: local magnetic susceptibility inhomogeneities induce local static magnetic field variations which in turn result in dephasing of the transverse magnetization proportional to the strength of the magnetic field alteration and proportional to the echo time during a gradient echo experiment. This local dephasing of the transverse magnetization results in increased signal cancellation within magnitude images when echo time increases (Fig 4A2, 4A3, 4B2 and 4B3 and in Fig 5F and 5G) and in local phase variations within gradient echo phase images (see example of high pass filtered phase images of the T2* weighted 3D datasets in Fig 5H).

### Ex vivo 3D spin echo images demonstrate that susceptibility inhomogeneities are paramagnetic

**Fig 6:**
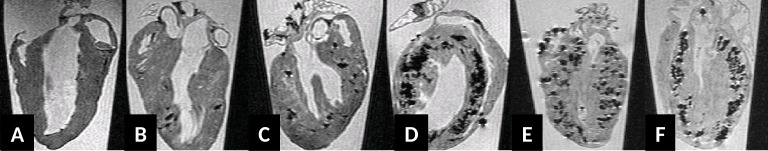
Evolution of the "paramagnetic susceptibility contrast" of CVB3 induced lesions as a function of time post infection as revealed by additional T2 weighted images (same imaging conditions as in Fig 5), (A) control animal, (B) SWR/J mouse 6d pi, (C) SWR/J mouse 9 d p.i., (D) ABY/SnJ mouse 14 d p.i., (E) ABY/SnJ mouse 18 d p.i., (F) ABY/SnJ mouse 8 weeks p.i.. Qualitatively, the paramagnetic contrast becomes clearly visible during acute myocarditis (B, C), increases at the subacute myocarditis (D, E) and remains very strong at all chronic stages of myocarditis (F).

For all infected animals showing T2*w hypointense contrast for which additional spin echo 3D datasets had been recorded, a hypointense patchy contrast was visible (Figs 5B and 6A–6D), which correlated perfectly with the hypointense contrast observed in gradient echo images. The contrast observed in spin echo experiment was strong and visible at all echo times but no T2 blooming effect was present. See example in Fig 5B (short TE) and Fig 5C (long TE). Non-infected animals showed homogeneous myocardium in T2 and T2* images as illustrated in Fig 5A and 5E. The apparent size of the tissue lesions in spin echo images appeared smaller in spin echo images with TE = 10 ms (Fig 5B) than in gradient echo images with TE = 3 ms (Fig 5F). This result strongly supports the hypothesis that the observed hypointense T2 and T2* contrasts originate mainly from strong local susceptibility inhomogeneities.

To assess the cause of the susceptibility differences observed between lesions and healthy tissue, the frequency encoding of the spin-echo images was recorded with a relatively "low" voxel bandwidth of 190 Hz/voxel and the resulting *ex vivo* spin echo heart images compared to spin echo images of air bubbles placed in water and measured with the exact same imaging parameters. Small, isolated regions of altered magnetic susceptibility (isolated air bubble, isolated blood clot or isolated lesion) are expected to behave like small magnet dipoles, creating dipolar patterns in gradient echo phase images. Such dipole patterns are visible in Fig 5H for some tissue lesions and in trapped air/blood clot (Fig 5A, colored inset). Those dipoles induce in spin echo images a characteristic arrow-like pattern as observed in Fig 5D for the air bubble phantom and in Fig 5B for tissue lesions. Those arrow-like patterns do point in the same direction for air bubbles and for isolated cardiac lesions. These results demonstrate that the regions with altered magnetic susceptibility within infected myocardium are paramagnetic with respect to (fixed) healthy tissue (or water) [16,17]. The paramagnetic character of the local magnetic field inhomogeneities was observed *ex vivo* in spin-echo images for diseased animals during the chronic phase of myocarditis but at earlier times points of myocarditis as well (Fig 6). Qualitatively, the arrow-like paramagnetic contrast is already clearly visible as early as 9 d p.i. (Fig 6C), becomes strong at day 14 p.i. (Fig 6D) and remains very strong at all other chronic stages (Fig 6E and 6F).

In correlation with the first histological indications of cardiac lesions (and necrotic myocytes) at 6 d p.i, T2* blooming and hypointense T2 contrast is visible at 6 d p.i. as well, but the geometry of the patterns is more difficult to interpret (Fig 6B) in terms of paramagnetic inhomogeneities. This might be due to the small size of the lesions at this time of observation, or to a less paramagnetically altered environment.

In very good correlation to those observations, Prussian blue stainings were always positive at all stages of disease, but appeared less prominent in cardiac lesions early in infection and were generally much stronger at day 14 p.i. and at later stages of the disease.

Taken all together, the *ex vivo* MRI observations reveal that the source of hypointense contrast in gradient echo images is paramagnetic. This rules out calcification as a main source of T2* contrast, since calcification is expected to be diamagnetic and strongly support the pathophysiological hypothesis that iron deposits, which are paramagnetic, are the source of observed T2*w and T2 hypointense contrast.

### Static magnetic field dependence

**Fig 7:**
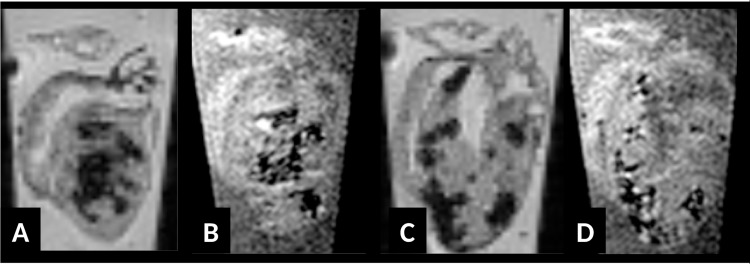
Comparison of long axis T2* weighted MRI images of two ABY/SnJ infected mouse hearts acquired at 17.6T (A and D) and 1.5T (B and D) with TE = 20ms and an isotropic resolution of 140um. Hypointense T2* contrast appears weaker at low magnetic field strength but is still clearly visible.

In Fig 7, the T2*w images of infected ABY/SnJ hearts with severe myocarditis are presented when measured at lower magnetic field strengths. The T2* hypointense contrast appeared much stronger at a very high magnetic field of 17.6T (Fig 7A and 7C) compared to a clinical magnetic field strength of 1.5T (Fig 7B and 7D). These observations are consistent with the hypothesis that iron deposits are the main source of T2/T2* contrast. Of translational interest is the observation that the T2*w contrast remained well observable *ex vivo* at 1.5T, suggesting that one will be able to diagnose *in vivo* CVB3-induced myocarditis in this animal model using human scanner.

### *In vivo* T2* MRI study of acute and chronic myocarditis at high magnetic field strength of 7T and 17.6T

**Fig 8:**
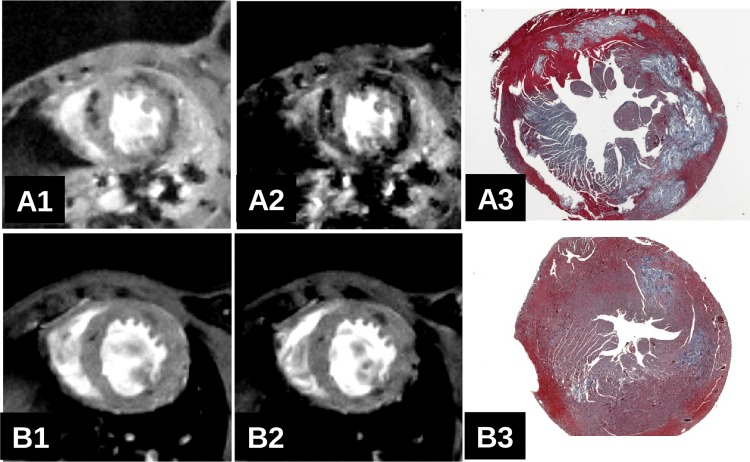
In vivo short axis T2*w heart images at 7T of two infected ABY/SnJ mice (A: 12 d.pi., B: 14 d.i.) using a short TE (A1, B1: TE = 2.5ms) or a longer TE (A2, B2: TE = 6.3ms) and Masson's trichrome histology (A3, B3). Note the T2* blooming effect on in vivo T2*w images with increasing echo time. The amount and location of the T2* hypointense regions correlates well with histology.

No adverse effect was observed during and after MRI investigations for all animals investigated. CVB3-infected mice with severe acute (Fig 8A3) or severe chronic (Fig 4A4 and 4B4) myocarditis were found to exhibit large hypointense regions within the myocardium in *in vivo* T2* weighted MRI (Fig 8A1 and Fig 4A1 and 4B1 and supplementary cine S3 and S4 Movies). In accordance with the previously presented *ex vivo* results, the location of the hypointense regions matches well with the lesions observed in histology. Accordingly, animals with a mild infection and a smaller extent of myocardial lesions (Fig 8B3) revealed fewer dark spots in T2* weighted images (Fig 8B1). In some hearts where only a few, very small lesions are present, MRI does not detect myocardial lesions. This can be explained by the higher sensitivity of histology which is capable to detect myocyte damage at the single cell level compared to MRI. Detailed results are presented in Tables 1 and 2. *In vivo*, a lesion specific T2* blooming effect was always observed as illustrated in Fig 8A1 (short TE, severe myocarditis) compared to A2 (long TE, severe myocarditis) and B1 (short TE, weak myocarditis) compared to B2 (long TE, weak myocarditis), demonstrating that local susceptibility inhomogeneities *in vivo* and *ex vivo* are the main cause of the observed contrast.

**Table 2:** Comparison of the MR / histology findings for all animals with disease 9dpi or later.

	*MR1*	*MR2*	*MR3*
***H1***	7	0	0
***H2***	7	8	0
***H3***	0	0	13

The abbreviations MR1, MR2 and MR3 are used to designate the 3 defined MR groups: MR1 no myocarditis, MR2 mild myocarditis and MR3 severe myocarditis. The abbreviation H1, H2 and H3 are used to define the histological groups no myocarditis (H1, observed only in control animals), mild myocarditis (H2) and severe myocarditis (H3).

### Visualization of inflammation using superparamagetic iron oxide nanoparticles

**Fig 9:**
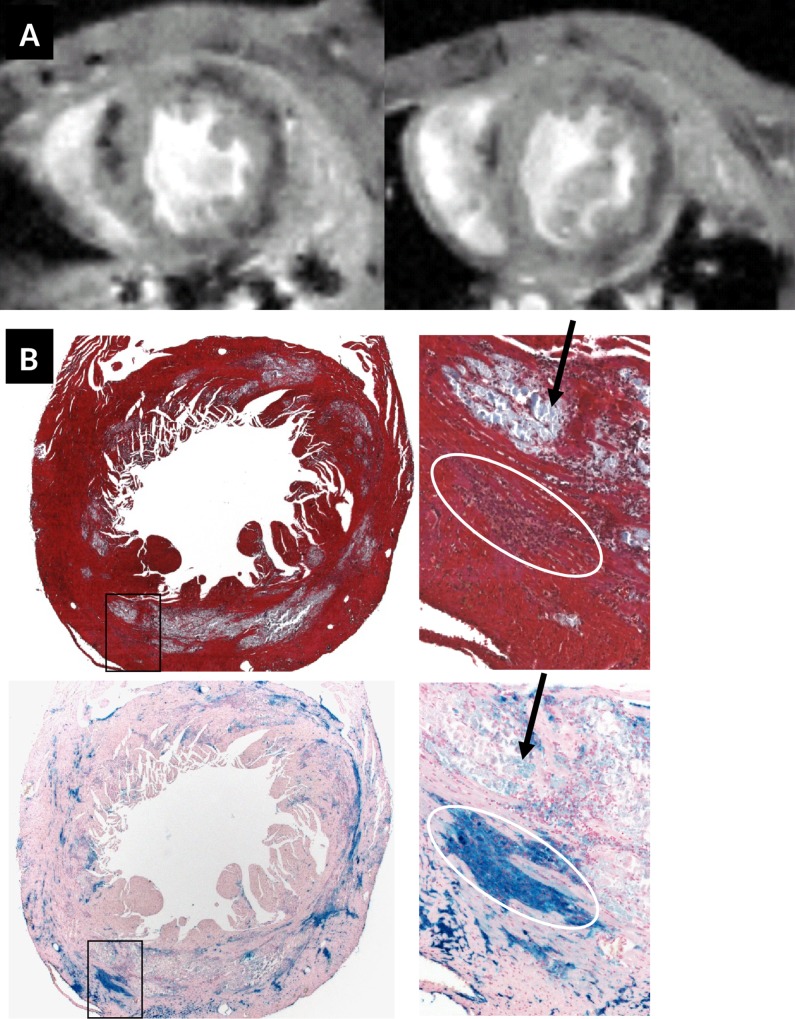
(A) short axis T2*w MRI heart image before (left) (same as Fig 8A1) and two days after ferumoxytol injection (right) of a CVB3 infected ABY/SnJ mouse (left, 12 days p.i., right 14 days p.i.) The endogenous MRI contrast due to CVB3 infection is clearly visible prior to ferumoxytol administration. After ferumoxytol administration the T2* hypointense contrast did not increase significantly. It is unclear whether ferumoxytol-containing macrophages or necrotic myocytes or both induce the hypointense contrast in T2*w images observed in Fig A (right). (B) A clear ferumoxytol uptake by macrophages was found 2 days after contrast agent injection and 14 days p.i., as demonstrated by Masson's trichrome (middle row, x12,5 and x200) and Prussian blue (bottom rows, x12,5 and x200) staining.

A further aim of our investigations was to evaluate whether cardiac inflammatory immune cells, especially macrophages, can be specifically detected by T2*contrast in CVB3-infected mice. For these experiments, we used different kinds of nanoparticles including the superparamagnetic iron oxide nanoparticles Feraheme™, revealing comparable results. Feraheme™ consists of ferumoxytol and was approved for iron replacement therapy in patients with anemia. This makes this MRI contrast agent [18,19] very attractive. ABY/SnJ mice were infected with CVB3 for 12 days and tail-injected with a 10mg Fe /kg ferumoxytol dosis. Animals were measured with MRI *in vivo* one hour prior ferumoxytol injection (Fig 9, top row, left image) and two days after (right image). It was interesting to realize that the hypointense T2* contrast of the severely inflamed myocardium did not significantly change before and after ferumoxytol injection.

A clearly enhanced ferumoxytol uptake by macrophages was found 2 days post contrast agent injection as demonstrated by Prussian blue staining (Fig 9, middle and bottom rows). However, so far it is unclear whether primarily ferumoxytol-containing macrophages or necrotic myocytes or both induce the hypointense contrast in T2*w images. Further MRI experiments are needed to investigate whether iron deposits in myocytes can be discriminated from induced iron storage in macrophages by this approach in order to provide a tool for the diagnosis of acute and chronic myocarditis.

## Discussion

In this study, we have shown that T2* MRI imaging is capable to detect virus-induced inflammatory cardiac lesions *in vivo* and *ex vivo* in different mouse strains at a very high resolution. Our data demonstrate that T2*weighted imaging is a very sensitive and reliable technique to diagnose *in vivo* the severity of myocarditis induced by CVB3 infection at different stages of the disease.

Correlation of *ex vivo* gradient echo and spin echo images with histology revealed that the hypointense areas in MRI reflect tissue lesions becoming paramagnetic in the course of infection. Prussian blue staining illustrates that paramagnetic areas in the heart are due to iron deposits. From former investigations, we know that CVB3-infected hearts are characterized by iron deposits in infected cardiomyocytes and some macrophages resulting from altered iron metabolism from day 6 p.i. until late stages of myocarditis [13]. Iron deposition is known to be paramagnetic which was shown in numerous MRI investigations. In absence of other known pathophysiological events in this animal model causing strong local paramagnetism within cardiac lesions and in absence of microbleeds, it can be concluded that iron deposits are the cause for the MRI contrast as observed at any stage of the CVB3-induced disease in ABY/SnJ and SWR/J mouse strains.

The existence of paramagnetic iron overload within virus-induced tissue lesions in this murine myocarditis model explains the visualization of tissues with very high sensitivity using only T2*w imaging avoiding injections of contrast agents as commonly used in Late Gadolinium Enhancement (LGE) CMR experiments. This is a remarkable finding in CVB3-infected mice. Whether iron overload is also present in heart tissue lesions of patients with enteroviral myocarditis has to be determined. In addition, it would be interesting to investigate whether humans with viral myocarditis reveal different T2*w imaging than patients with e.g. non-infectious myocarditis.

To the best of our knowledge, no hypointense T2* contrast have been reported in humans with myocarditis yet. However, disturbances in iron metabolism might be associated with the involvement of inflammatory heart diseases). Recently, we reported a patient with iron overload in association of various cardiac virus infections (HHV6, EBV and PVB19) and inflammation [20]. So far, the interrelationships between iron, viral infections and immune reactions are not well studied. It is suggested that high iron concentrations might increase the susceptibility for infections by modifying the host immune response, specifically by impairment of cell-mediated immune mechanisms. E.g., increased loading of macrophages with iron was found to result in the inhibition of IFN-gamma-mediated pathways. In hepatitis B infection, it was described that patients with higher levels of serum iron or ferritin are less likely to achieve spontaneous recovery after acute HBV infection. It is also reported that excess iron enhances fibrogenic pathways and worsens the clinical course of HCV infection by causing oxidative stress. In addition, excess iron is capable to decrease the viability of HIV-infected cells and elevates the activity of reverse transcriptase, indicating that iron overload associated with HIV infection is detrimental to host cell responses against this infection [21]. More recently, we found that iron overload in the myocardium may potentiate the effects of enterovirus infection by the NO/HO-1 pathway, thus increasing cardiac pathogenicity by oxidative stress [13].

By electron microscopy iron deposits seem to be mainly located in structurally affected mitochondria of infected myocytes. This observation explains well the finding that the necrosis of myocytes in the course of CVB3 infection is associated with a diminished energy metabolism as suggested by Schulze et al. [22]. In their paper a tight correlation was described between a disturbed energy turnover and the cardiac performance in CVB3-infected ASW/SnJ mice, confirming the association of mitochondrial dysfunction with the pathophysiology of the disease. A close correlation between high cardiac CVB3 titers with cardiac mitochondrial dysfunction was demonstrated in susceptible ASW/SnJ mice during acute myocarditis [23]. Thus, the imbalanced energy levels due to disturbances in iron metabolism contribute to the severity of the enteroviral myocarditis in mice. Importantly, perturbations in energy metabolism has not only been demonstrated in murine but also in human enteroviral myocarditis [24], assuming that related processes might contribute to heart failure in chronic myocarditis.

The value of the study is limited by the absence of Gadolinium late enhancement imaging during acute and chronic myocarditis. LGE provides MR contrast related to fibrosis or lesion volume and it would be of great interest in the next study to compare both contrasts to get valuable insights into the interplay between the two different sources of contrast.

In the imaging community, there is much interest in visualizing inflammation processes in the heart by using MRI using cellular and molecular MR imaging strategies based on the administration and detection of contrast agents designed to induce local susceptibility artifacts and therefore hypointense T2* contrast. However, as shown in our study, such strategies might suffer from low sensitivity and reduced/complicated diagnostic value since it might become very difficult to differentiate between exogenous and endogenous magnetic susceptibility induced T2* signal losses. This is a severe problem enhanced by the fact that most probably the location of the targeted molecular events i.e. for the visualization of immune cells will correlate spatially with the cardiac lesions characterized by the endogenous T2* contrast. Furthermore, the endogenous T2* contrast allows *in vivo* to localize a lesion with good sensitivity, but the resolution dependent blooming nature of T2* contrast makes lesion appear bigger than they are in reality, increasing the possibility of image contrast interference between exogenous and endogenous T2*w contrast. To illustrate this problem, we reported on an experiment we have been conducted on the group of CVB3-infected animals measured *in vivo* at day 14 p.i. (Fig 9). In this experiment, the endogenous hypointense T2* contrast appeared rather unchanged two days after ferumoxytol injection, and it was not possible to spatially differentiate hypointense regions (Fig 9, top right) resulting from iron deposits in necrotic myocytes from labeled macrophages after uptake of iron-containing nanoparticles.

In future, it is feasible to think of using the strong R1-relaxivity properties of iron oxide contrast agents and try to implement ultra-short echo time based protocols to avoid the contrast interference between accumulated endogenous and extraneous paramagnetic substances. However, there is an additional physiological dilemma resulting from the administration of iron oxide contrast agents with pathological iron overload: the biodegradability of some iron oxide contrast agents like ferumoxytol can be very high, resulting in an increased plasma iron concentration. This high level of plasma iron might lead to further incorporation of iron in infected myocytes, resulting first in a potential increase of the endogenous T2* and second in a possible modulation of the pathology of affected myocytes subjected to iron overload. On the other hand, we have shown that high concentrations of ferumoxytol (40mg Fe) correlates with strong Prussian blue stainings (Fig 9), not only of necrotic myocytes but also for infiltrating macrophages, suggesting that inflammation might be visualized using ferumoxytol in this animal model. Further MRI investigations are needed to investigate whether it is possible to differentiate iron positive myocytes from infiltrating macrophages invading the heart during infection. Also, it will be important to see whether by this approach patients with infarction can be differentiated from patients with myocarditis. Yilmaz et al. [25] demonstrated that ultrasmall superparamagnetic iron oxide nanoparticles (USPIO) enable a more detailed characterization of myocardial pathology mainly by detecting infiltrating macrophages in patients with myocardial infarction.

A very promising alternative to imaging inflammation with iron oxide contrast agents in presence of local susceptibility inhomogeneity is given by ^19^F MRI. Ye et al. [26] have shown experimentally that ^19^F MRI can be a very robust MRI imaging technique in regions with strong susceptibility inhomogeneities. Recently, the experimental application of ^19^F MRI to image the infiltration of macrophages in the murine model of CVB3 myocarditis was presented [10,11].

## Conclusion

In conclusion, we show in this study that iron deposition resulting from disturbed iron metabolism in necrotic myocytes of cardiac lesions of CVB3-infected mice results in a strong magnetic susceptibility difference between lesions and healthy myocardium which in turns results in strong T2* contrast *in vivo* and *ex vivo*. This MRI technique provides a fast and sensitive diagnostic tool to determine the severity of viral myocarditis and the precise localization of tissue damage without the use of any contrast agents in this model system.

## Supporting information

S1 Movie *Ex vivo* T2*w 3D animated vizualization of cardiac lesions in a ABY mouse 9 d.p.i. at 17.6T. (MPG)

S2 Movie *Ex vivo* T2*w 3D animated vizualization of cardiac lesions in a ABY mouse 14 d.p.i. at 17.6T with 20um resolution. (MPG)

S3 Movie Vizualization of cardiac lesions in a short axis Cine movie of an infected ABY mouse at 17.6T. (AVI)

S4 Movie Vizualization of cardiac lesions in a short axis Cine movie of an infected ABY mouse at 17.6T. Higher spatial resolution.
(AVI)

S5 Movie Vizualization of cardiac lesions in a long axis Cine movie of an infected ABY mouse at 17.6T. (AVI)
